# Recent insights into α-carboxysome structure, mechanism, and assembly

**DOI:** 10.1128/jb.00366-25

**Published:** 2026-01-12

**Authors:** Samuel L. Hartzler, Kristy Rochon, Samstita Laxminarayan Raja, Lauren Ann Metskas

**Affiliations:** 1 Department of Biological Sciences, Purdue University 311308 , West Lafayette, Indiana, USA; 2 James Tarpo Jr. and Margaret Tarpo Department of Chemistry, Purdue University 311308 , West Lafayette, Indiana, USA; University of Southern California, Los Angeles, California, USA

**Keywords:** BMC shell proteins, csoSCA, csoS2, cso operon, rubisco, carboxysomes, bacterial microcompartments

## Abstract

Bacterial microcompartments (BMCs) are pseudo-organelles that sequester metabolic enzymes, intermediates, and/or gases within the bacterial cytosol. One model BMC is the carboxysome (CB). CBs facilitate rubisco-driven fixation of CO_2_, increasing efficiency and maximizing the phosphoglycerate output in CB-containing bacteria. The α-CBs are of particular interest due to their small size and relative simplicity, making them ideal targets for bioengineering applications. These CBs were the first BMC observed and have been a long-studied model; however, they are challenging to study in native systems and in purified samples. Recent advances in cryogenic electron microscopy and cryogenic electron tomography have resulted in many new published structures of the shell proteins, shell assemblies, and cargo organization within the CB. These new insights have advanced the field’s understanding of important structural interfaces, shed insights into once unknown domain functions, and the complex mechanisms involved in assembly and maintenance of the CB. This review highlights recently published structures of α-CB proteins and the functional and mechanistic findings of these studies.

## INTRODUCTION

**Fig 1:**
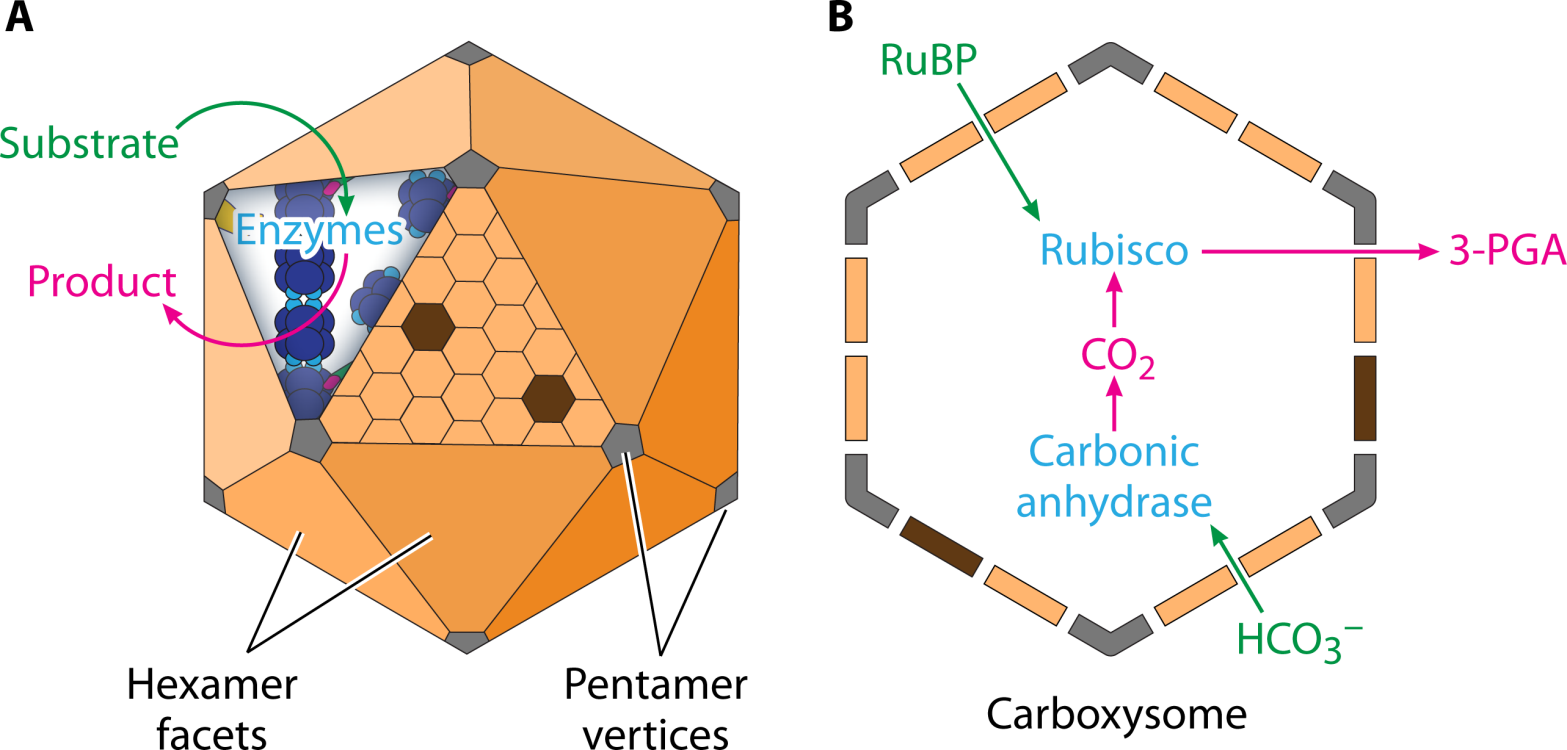
Bacteria microcompartment organization. (**A**) All BMCs share homologous shell proteins that self-assemble to form icosahedral protein structures within prokaryotic cells. Substrates and products diffuse across the shell, while enzymatic proteins are sequestered inside. The enzyme cargo is typically a piece of a metabolic pathway and varies according to the specific BMC. Products of these enzymatic pathways exit the shell for use in cellular pathways. (**B**) The model BMC is the carboxysome (CB), an anabolic system that converts carbon dioxide to 3-carbon sugars by the rubisco enzyme.

Compartmentalization is a repeated cellular motif that allows optimization and regulation of essential enzymatic life functions. In contrast to the membrane-bound organelles of eukaryotes, prokaryotes utilize protein-based complexes to compartmentalize these reactions (1). Bacterial microcompartments (BMCs) are self-assembling polyhedral protein shell compartments encapsulating various metabolic enzyme cargos (2) (Fig. 1A). Despite being widely distributed among prokaryotes (3, 4), BMCs are difficult to study in their native state as they are functionally diverse (3), structurally heterogeneous (5), and challenging to purify (6).

The encapsulated cargo of a BMC determines its function, typically anabolism (CBs, Fig. 1B) or catabolism (3). The CB has become a model BMC due to its constitutive expression under laboratory conditions (7) and its simplicity relative to other known BMCs (3). CBs encapsulate rubisco (carbon fixation) (7) and carbonic anhydrase (bicarbonate to carbon dioxide interconversion) (8), functioning as part of a carbon-concentrating mechanism (CCM).

CCMs are a broad category of diverse strategies that are used by various plants and bacteria to increase the efficiency of carbon fixation (9). CBs increase the local concentration of carbon dioxide around the rubisco (10) while minimizing the competing oxygen-mediated photorespiration activity (11). Inorganic carbon transporters bring bicarbonate into the cell to create a cytoplasmic carbon pool (12, 13), driving diffusion of bicarbonate across the CB shell (14). Within the CB lumen, the carbonic anhydrase catalyzes the interconversion between bicarbonate and CO_2_ (15), and the rubisco fixes the carbon to convert CO_2_ into 3-phosphoglycerate for use in downstream metabolism (16).

While CBs conserve these CCM core elements, they diverge in morphology and sequence and are therefore further classified into alpha (α) and beta (β) CBs. α-CBs express the genes for the CB at a single operon, contain form-IA rubisco and a beta-class carbonic anhydrase, are smaller, and are expressed by α-cyanobacteria and chemolithotrophs in stable aquatic environments (17–19). β-CBs express the genes for the CBs at multiple gene clusters, contain form-IB rubisco and a gamma-class or beta-class carbonic anhydrase, are larger, and are primarily expressed by freshwater β-cyanobacteria in rapidly fluctuating environments (17–19). Additionally, α- and β-CBs assemble through diverging mechanisms (20): α-CBs assemble through simultaneous aggregation of rubisco, shell, and the scaffolding protein CsoS2 (21–24), while β-CBs assemble rubisco paracrystalline arrays before recruiting shell proteins with the scaffolding protein CcmM and chaperone CcmS (25–27).

α-CBs are of particular interest in bioengineering because they can be readily purified (7) and have been successfully reconstituted in a heterologous system (28). However, because β-CBs are more accessible with light microscopy methods (29, 30), they are better understood for cellular interactions and assembly. Despite this, β-CBs remain difficult to purify for structural studies, possibly a result of their large size and flexible properties (31, 32); therefore, structure and functional studies *in vitro* are typically carried out in α-CBs, while β-CBs are frequently used to study *in vivo* assembly (32). In recent years, advances in structural biology methods have allowed the field to expand its structural understanding of α-CBs. This review will highlight recent structural advances within α-CBs that contribute to functional understandings of these structures.

## CSO OPERON

**Fig 2:**
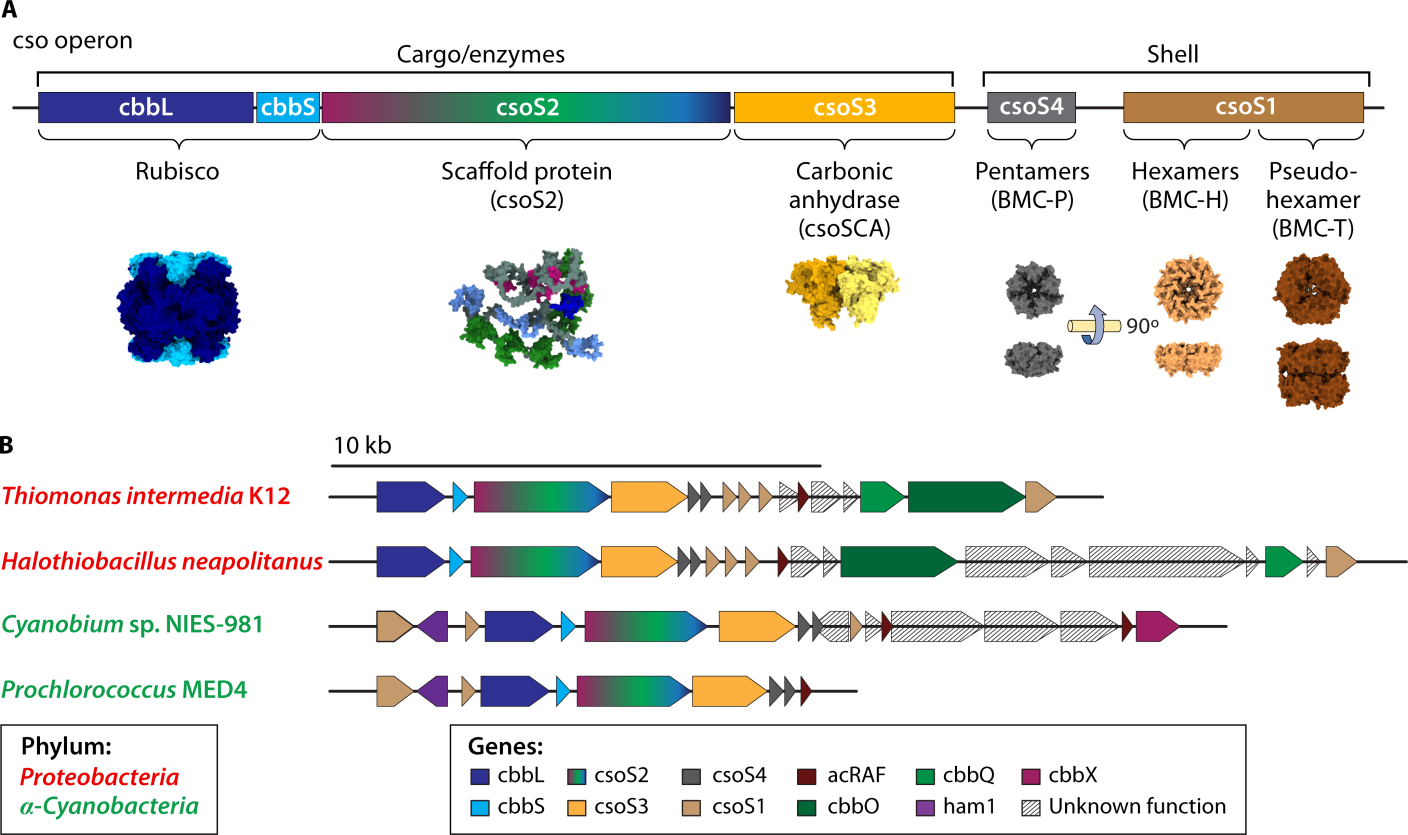
cso operon locus in selected α-CBs. (**A**) The cso operon can be divided generally into cargo/enzymes and shell. The cargo/enzymes include CbbL, CbbS, CsoS2, and CsoSCA, which occur with approximately 450:400:450:60 functional copies, respectively, per α-CB (35). The CbbL and CbbS together assemble the rubisco (pictured PDB ID: 6UEW) (22); the CsoS2 (pictured AlphaFold model: AF-O85041) (36, 37) acts as a scaffold; and CsoS3 encodes the carbonic anhydrase (pictured PDB ID: 2FGY) (38). There are multiple genes expressing shell proteins; however, each α-CB requires a pentamer (CsoS4, pictured PDB ID: 2RCF) (39) to form the vertices and hexamers (CsoS1, pictured PDB ID: 3H8Y) (40) to form the facets. Pseudo-hexamers (CsoS1D, pictured PDB ID: 7DHQ) (41) have been observed to form from a dimer of trimers. These shell proteins (CsoS1:CsoS4:CsoS1D) occur with approximately 1,000:11:3 functional copies per α-CB (35). (**B**) Adapted from Zhou et al. 2024 (24), cso operons and surrounding regions from organisms expressing α-CBs whose recent structures have been published and highlighted in this review. The cso operon has been found in both proteobacteria and α-cyanobacteria. *Prochlorococcus* MED4 is considered the most basic operon (24).

Proteobacteria and α-cyanobacteria express canonical α-CB genes at a single gene cluster, the cso (**C**arboxy**SO**me) operon (18, 33). The cso operon expresses key structural components of the CB: the small and large form IA rubisco subunits (CbbL and CbbS), a beta-class carbonic anhydrase (CsoSCA), scaffolding protein(s) (CsoS2), and numerous shell proteins (CsoS1 and CsoS4), with an additional shell protein (CsoS1D) conserved nearby (24) (Fig. 2A). These genes are differentially expressed and translated (33, 34). The shell proteins CsoS1, CsoS1D, and CsoS4(A/B) contain homologous “BMC fold” domains, which form hexameric (BMC-H), pseudohexameric (BMC-T), and pentameric (BMC-P) structures, respectively, to make the facets and vertices of the pseudo-icosahedral shell (2). The reliance on BMC domains and compositional heterogeneity is conserved across all known BMC shells (3).

The cso operons diverge between organisms in sequence arrangement (24), in the presence/absence of multiple paralogs of shell proteins (24), or the presence/absence of a short form of the CsoS2 scaffolding protein (42). Together, these factors are responsible for the substantial variation between cso operons (24) (Fig. 2B). The regions around the cso operon also diverge with the presence/absence of additional “peripheral” proteins, which are increasingly implicated in α-CB function, including a rubisco activase (43, 44), a putative rubisco chaperone (45), a ParA-type ATPase (46), an inorganic carbon transporter (13), and proteins with an unknown function (24) (Fig. 2B). Finally, numerous additional loci that retain elements of the cso operon have been identified, either representing repurposed or vestigial remnants of CB loci (47).

## GLOBAL ORGANIZATION

The polyhedral structure of purified α-CBs was first observed with electron microscopy (48, 49). Recombinant expression of BMC shells combined with increasingly high-resolution imaging capabilities has since yielded several models for spatial localization of the different shell proteins, providing potential mechanisms for nonplanar interactions that form the icosahedral symmetry in generic shell BMCs (21, 41, 50, 51), as well as potential anchoring sites for scaffolding of internal enzymes (21). These findings are supported by the conservation of these residues, particularly along the hexamer-pentamer interface (21, 41, 52).

Besides the shell layer, these studies also identified layers of internal densities corresponding to the encapsulated enzymes in α-CBs (24, 49, 53). The function of α-CBs was first suggested by the discovery of rubisco inside the polyhedral bodies of *Halothiobacillus neapolitanus* using electron microscopy (7). Subsequent *in vitro* and *in situ* cryo-electron tomography (cryo-ET) has revealed additional features of unknown significance, including partial or broken shells (5, 54, 55), elongated or abnormal morphologies (5, 55), cytoplasmic rubisco aggregates (54), and inclusion bodies and non-rubisco densities inside the α-CBs (5, 55).

## SHELL

### Hexamer (CsoS1)

**Fig 3:**
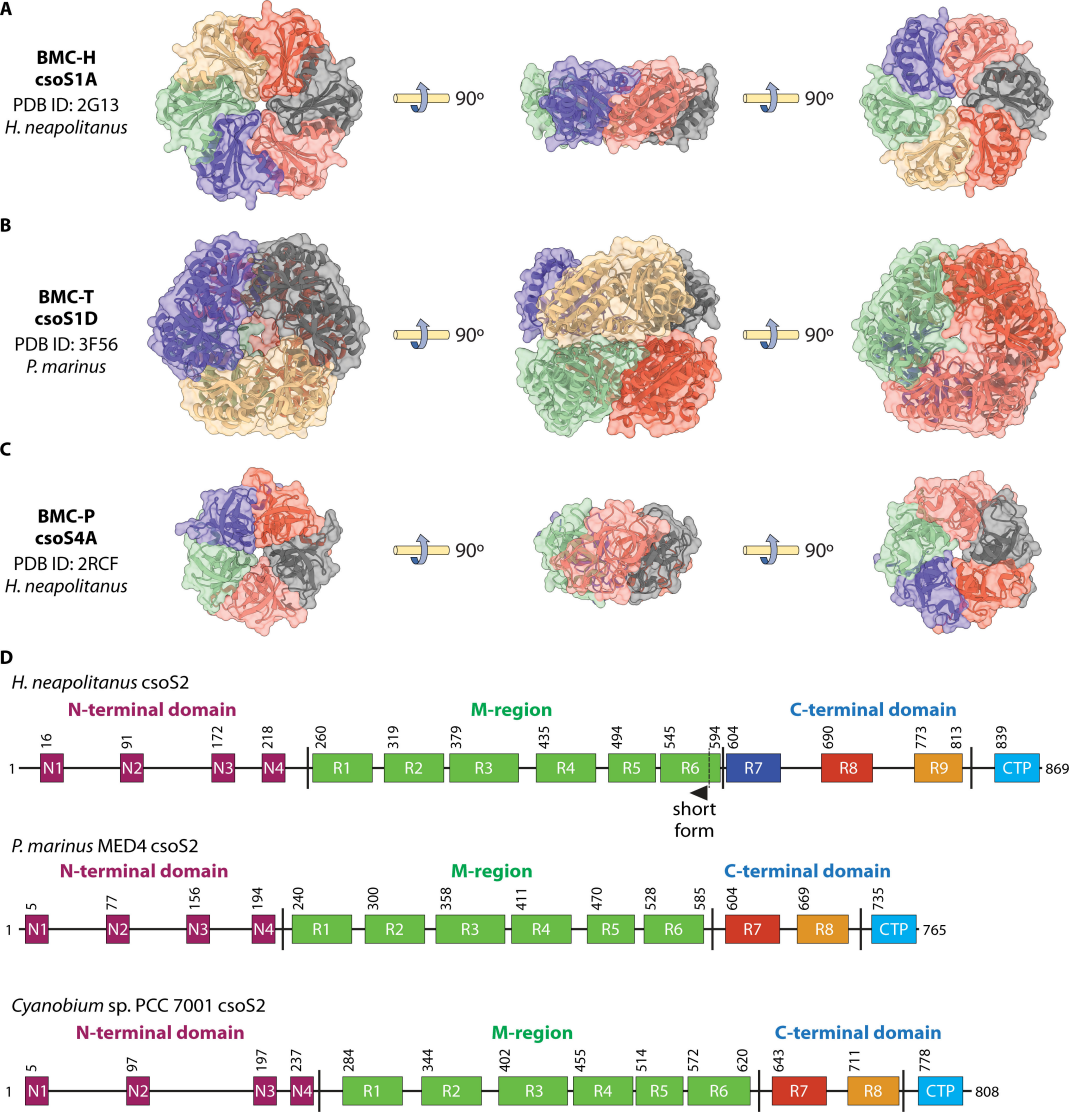
Structural components of the α-CB shell. (**A**) Representative hexamer structure from *H. neapolitanus* (PDB ID: 2G13) (57). (**B**) Representative pseudo-hexamer structure from *P. marinus* MED4 (PDB ID: 3F56) (64). (**C**) Representative pentamer structure from *H. neapolitanus* (PDB ID: 2RCF) (39). (**D**) Differences in the CsoS2 sequence between organisms. *H. neapolitanus* expresses a short form and a long form mediated by a frameshift at the R6 repeat in the middle domain (65). *P. marinus* MED4 (24) and *Cyanobium* PCC-7001 (data accessible at NCBI Protein, accession WP_043369500) express a single CsoS2 isoform that contains one less C-terminal repeat compared to the *H. neapolitanus* CsoS2B.

The BMC-H domain-containing CsoS1 was the first α-CB shell protein to be identified (56) and structurally characterized as a BMC-fold protein forming flat hexamers (57) (Fig. 3A). These hexamers are likely composed of heterogeneous combinations (21) of several BMC-H paralogs that diverge only at the C terminus (24, 57). The hexamers localize to the shell facets and may facilitate substrate and product translocation (57, 58). Hexamers of CsoS1 contain a central positively electrostatic pore (~4 Å) hypothesized to facilitate diffusion of small negatively charged particles such as bicarbonate (57, 58). While numerous studies have suggested that the BMC-H pore (59, 60) and the full shell structure (61, 62) have reduced permeability for gases such as O_2_ and CO_2_, recent modeling suggests that the shell is still highly permeable to CO_2_ (63); more work is needed in this area.

### Pseudo-hexamer (CsoS1D)

The BMC-T domain in BMCs is characterized by a tandem repeat of two BMC domains that form pseudohexamers in solution with a central threefold axis of symmetry (64) (Fig. 3B). While this domain was first discovered in α-CBs (64), it was later shown to be incorporated across a broad diversity of BMC structures (52). α-CBs contain a single ortholog of this domain, *csoS1D* (64). While this gene is separated from the rest of the cso operon of some α-CBs, the theorized presence of this off-site gene in the α-CB (64) was later supported by the detection of CsoS1D in purified α-CBs (66), incorporation in heterologous α-CBs (67), and additional structural modeling (41). On average, three copies of CsoS1D pseudohexamers are present in purified α-CBs (35).

CsoS1D contains a large (~14 Å) pore at the center of the pseudohexamer (64). This pore could adopt an “open” or “closed” conformation, mediated by the movements of conserved arginine and glutamine residues around the threefold axis (64). This morphology suggests an allosterically regulated mechanism for movement of larger metabolites across the shell (64), though the maximal flux would be restricted by the low copy number of these subunits. The CsoS1D crystal contained stacked dimers of closed and open trimers in solution, a morphology that has been observed in a recombinant BMC shell (68) but has not yet been identified in a native α-CB (64).

This structure of CsoS1D leaves several unanswered questions. The physiological role of the stacked CsoS1D structure remains unclear, raising the possibility that this is a transient interaction (2). Mechanisms such as allosteric regulation, which modulate the open and closed conformations, remain unknown (64), despite the presence of ligand-binding sites in BMC-T domains in BMCs such as β-CBs, which allosterically regulate pore opening (52, 69). While β-CBs contain diverging BMC-T paralogs (70, 71), α-CBs contain only a single (64) BMC-T ortholog that diverges between proteobacteria and α-cyanobacteria (3, 52). Finally, the location of the BMC-T pseudohexamer within the α-CB shell remains unclear, with conflicting suggestions that it is either randomly included in the facet (53) or localizing along the twofold axis of symmetry to introduce angles (50, 51).

### Pentamer (CsoS4)

The solved structures of CsoS4 proteins from the α-CBs of *Halothiobacillus neapolitanus* provided significant insights into the shell arrangement of the α-CBs (39). These proteins form a pentamer of monomers with cytosolic-facing concave and luminal-facing convex faces and a central ~2.9 or ~3.5 Å pore (39, 72) (Fig. 3C). As pentamers are necessary to form an icosahedron or other polyhedron, the discovery of a pentamer in the α-CB shell was a significant advance in understanding shell organization (39). This led to a refined model of the α-CB as an assembly of hexameric subunits with pentameric vertexes of CsoS4 paralogs (39). Several studies have identified pentamer/hexamer interactions from recombinant or purified α-CBs (24, 41, 73), and expression studies have confirmed that pentamers are expressed at low levels expected for incorporation into vertices (34, 62).

The two paralogs of CsoS4 (CsoS4A and CsoS4B) from *H. neapolitanus* are almost identical in sequence and mass but are experimentally differentiated by their isoelectric point (62). Structurally, these paralogs have similar secondary structures but contain diverging cytosol and luminal loop conformations in addition to different pore electrostatics (62). Recombinant expression of α-CBs suggests that these coexist in heteropentamers, the significance of which is poorly understood (21). Possible metabolite or enzyme interactions with the CsoS4A and CsoS4B pentamers are unknown, despite proposed interactions with the encapsulated rubisco and CsoS2, respectively (72).

Functionally, CsoS4AB mutants contain higher numbers of elongated α-CBs and require high CO_2_ concentrations for growth (62). However, while the elongated mutant α-CB phenotype is consistent with the model of hexameric facets and pentameric vertices, the mutants still formed occasional α-CBs similar in morphology to that of wild-type α-CBs, in that they contained distinct vertices (62). This leaves it unclear whether these morphologies could have functioned normally alongside the fully inactive elongated α-CBs and whether α-CB vertices must be solely pentameric.

### Shell assembly and cargo scaffolding (CsoS2)

The intrinsically disordered accessory protein CsoS2 is believed to play spatially separate roles in shell nucleation and cargo condensation during α-CB assembly (21–24), a unique assembly process and distinct from the cargo-first assembly model of β-CBs (25–27). Importantly, CsoS2 sequences diverge between taxa (Fig. 3D), providing possible mechanisms for the different morphologies observed. In several proteobacteria such as *H. neapolitanus*, CsoS2 is known to undergo a ribosomal frameshift to truncate the C-terminal sequence in roughly half the expressed protein (CsoS2A short isoform and CsoS2B long isoform) (65, 74–76). In contrast, the CsoS2 proteins of the α-cyanobacteria *Prochlorococcus marinus* (24) and *Cyanobium* sp. PCC 7001 (42) does not undergo this ribosomal frameshift. These differences in CsoS2 are thought to drive morphological differences in size and cargo packing between α-CBs from these taxa (23, 24, 42, 55).

Despite being largely disordered, CsoS2 has three main regions with conserved elements (74) (Fig. 3D): the N-terminal domain that organizes rubisco through conserved R RR GK repeats (22, 74), the middle region (M-region) with conserved KV VTG VTG C VTG Y C repeats that interfaces with the shell (23, 74, 77, 78), and the C-terminal domain with conserved VTG triplets that intercalate between shell proteins (C-terminal repeats) (23, 73, 74), concluding with a final C-terminal peptide, which was initially suggested to be externalized (74) but was subsequently resolved inside the shell (73).

Recent studies mutating the middle region repeats have highlighted the role of CsoS2 as a key regulator of α-CB shell assembly and size (23, 73, 74). Two recent CsoS2 studies were able to alter the shell size by modifying the number of middle region repeats (23) or deleting combinations of N-terminal region, middle region, and the C-terminal region (78). α-CB diameter of both *H. neapolitanus* purified and heterologously expressed α-CBs was found to increase with the number of middle region repeats, with the smallest α-CBs from constructs with only the N- and C-terminal domains (23). In parallel, heterologously expressed α-CB populations with CsoS2 lacking the middle region repeats contained both smaller-diameter particles with vertices and elongated particles that lacked the facets of wild-type α-CBs (78). As a result, the middle region repeats were suggested to provide the interactions with the shell hexamers needed to determine the curvature (78). However, a previous *in situ* study of wild-type *H. neapolitanus* α-CBs documented a similar small population of elongated α-CBs (55), suggesting that this model of CsoS2 middle region repeat-controlled curvature is incomplete.

The clearest information available on C-terminal CsoS2 interactions with the shell comes from several recent structural studies using recombinant shell assemblies of CsoS2 and hexamer and pentamer paralogs (41, 73). While wild-type CsoS2 from *H. neapolitanus* was additionally used by the 2023 paper (73), both studies mutated the C-terminal region of CsoS2 to generate small icosahedra that are more amenable to classical structural biology approaches (41, 73). This approach, while non-native, was needed to accomplish the resolutions necessary for the identification of key interfaces between shell proteins and shell-cargo interfaces.

**Fig 4:**
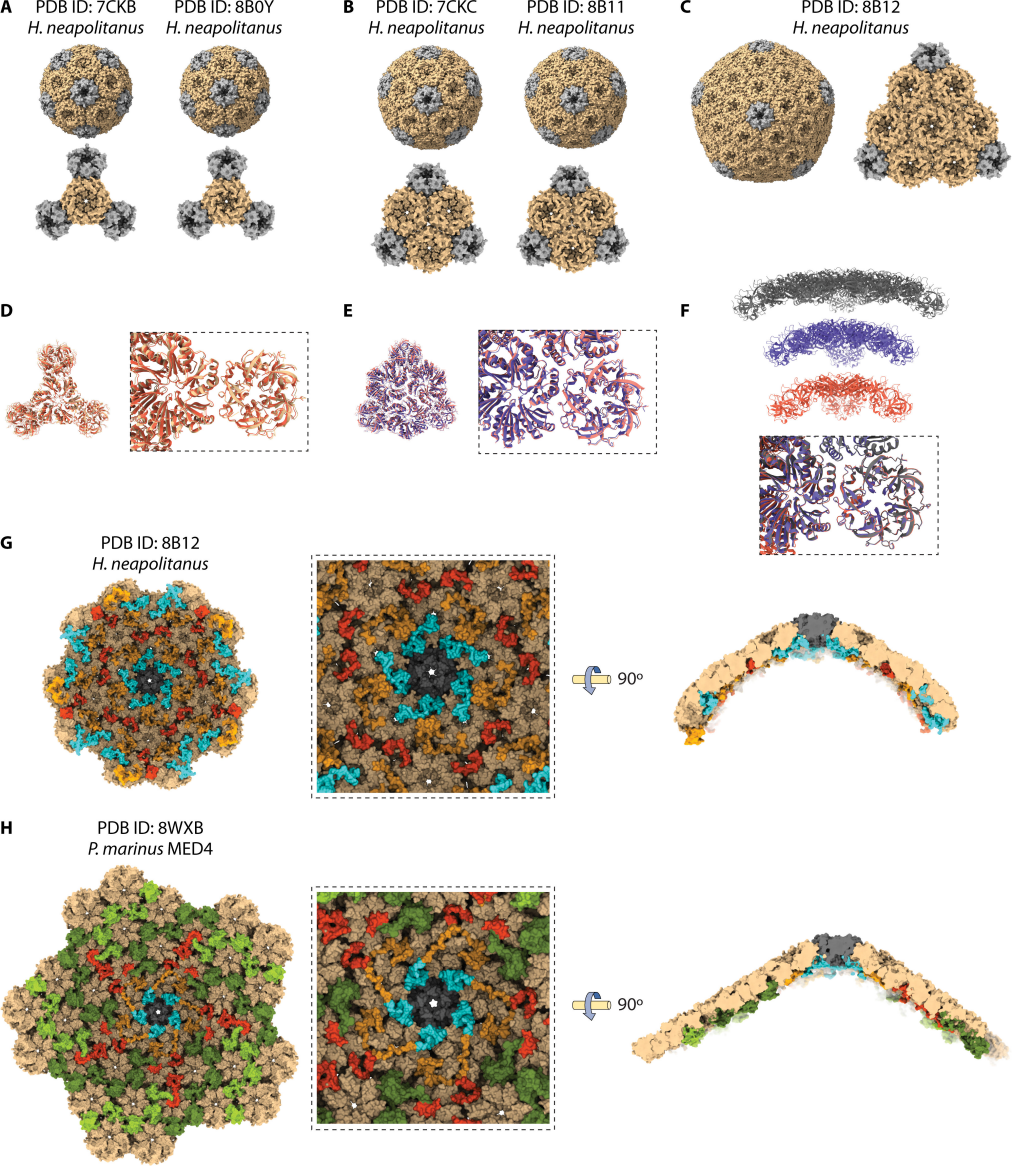
Mini-shell assemblies and larger CsoS2-stabilized shell structures derived from α-CBs provide insights into shell structure. (**A**) T = 3 mini-shell assemblies from *H. neapolitanus* (PDB ID: 7CKB) (41) and (PDB ID: 8B0Y) (73). (**B**) T = 4 mini-shell assemblies from *H. neapolitanus* T = 4 (PDB ID: 7CKC) (41) and (PDB ID: 8B11) (73). (**C**) T = 9 mini-shell assembly from *H. neapolitanus* (PDB ID: 8B12) (73). (**D**) T = 3 atomic structures (7CKB tan, 8B0Y orange) were aligned to the central hexamer, and small heterogeneity was observed in the pentamer’s position. (**E**) T = 4 atomic structures (7CKC pink, 8B0Y blue) were aligned to the central hexamer, and small heterogeneity was observed in the pentamer’s position. (**F**) Comparison of the T = 3 (orange, PDB ID: 8B0Y), T = 4 (blue, PDB ID: 8B11), and T = 9 (gray, PDB ID: 8B12) curvature and atomic structure alignments from Ni et al., 2023 (73). The expansion of the facet is accommodated by increasing curvature, while the pentamer and hexamer interface is unchanged. (**G**) Internal view of T = 9 structure centered on the pentamer vertex (PDB ID: 8B12) (73). CsoS2 fragments are as identified F1 (red), F2 (yellow), and F3 (cyan). A zoomed-in view is presented in the center. The right represents a central slice view of the side centered on the pentamer to demonstrate the curvature. (**H**) Internal view of T = 49 structure centered on the pentamer vertex (PDB ID: 8WXB) (24). CsoS2 fragments are identified as F1 (red), F2 (yellow), and F3 (cyan) along with the middle region repeats (green). A zoomed-in view is presented in the center. The right represents a central slice view of the side centered on the pentamer to demonstrate the curvature.

The first of these studies used cryo-electron microscopy (cryo-EM) to solve the structures to high resolution of miniature shells with T = 3 (Fig. 4A, EMD-30384, PDB ID: 7CKB) and T = 4 (Fig. 4B, EMD-30385, PDB ID: 7CKC) icosahedra, prepared with CsoS4A pentamers, CsoS1A hexamers, and a truncated CsoS2 C-terminal peptide (41). Subsequently, these mini-shells were later constructed with the same shell protein paralogs but with full-length CsoS2 or no CsoS2 (73). In this case, removal of CsoS2 resulted primarily in smaller T = 3 assemblies (Fig. 4A, EMD-15798, PDB ID: 8B0Y), while full-length CsoS2 resulted in primarily larger T = 4 (Fig. 4B, EMD-15799, PDB ID: 8B11) and T = 9 assemblies (Fig. 4C, EMD-15801, PDB ID: 8B12) (73). These assemblies provide insights into the shell interfaces between the subunits. Alignment of these T = 3 (Fig. 4D) and T = 4 (Fig. 4E) assemblies reveals some heterogeneity in the position of the pentamer (41, 73). Comparison of the T = 3, T = 4, and T = 9 structures from reference 73 reveals that the increasing curvature does not alter the interface between the pentamer and hexamer (Fig. 4F).

The resolution of the T = 9 structure was sufficient to identify ordered regions of CsoS2, all in the repeats in the C-terminal portion: the fragment (F)1 (R712-R731) and F2 (L773-G823) C-terminal repeats and F3 (E829-G869) C-terminal peptide. F1 and F2 formed interfaces with three hexamers, while the F3 C-terminal peptide formed interfaces with one pentamer and two hexamers (73) (Fig. 4G).

These CsoS2 interactions with shell proteins were also observed in a wild-type cryo-EM structure of the *Prochlorococcus* α-CB (24) (Fig. 4H). The shell vertex (T = 49) was resolved to 4.2Å (EMD-37902, PDB ID: 8WXB), high enough to identify CsoS2 regions but not shell paralogs (24). The authors also resolved shell interactions with the CsoS2 middle region for the first time (24). Comparing these interactions with those observed in the T = 9 mini shell (50, 73), the C-terminal peptide interfaces at the vertex are similar, but the wild-type shell has fewer interactions between the hexamers and the C-terminal peptide, and some C-terminal fragment interfaces with the hexamers also differ (24). These differences are possibly a result of reduced occupancy in wild-type α-CBs or differences in the curvature and facet size. These studies together support that certain C-terminal CsoS2 interactions are involved in shell formation and that the flexibility of the protein and promiscuity of its interactions may facilitate the structural heterogeneity of the full α-CB assemblies.

## ENZYMES

Two cargo proteins are found within the α-CB: the enzymes rubisco (CbbL_8_ and CbbS_8_) (7) and β-carbonic anhydrase (CsoSCA) (8). In addition, the previously discussed structural protein CsoS2 also interacts with the cargo as well as the shell to facilitate assembly (22). These proteins are encoded on the cso operon and expressed in a 13:1:2:2.5 stoichiometry of rubisco:CsoSCA:CsoS2A:CsoS2B in purified *H. neapolitanus* α-CBs (74).

### Signature enzyme (rubisco)

Rubisco sequestered in α-CBs are hexadecamers comprising eight large subunits and eight small subunits (79, 80). The active site is formed at interfaces between two large subunits in the core of the oligomer (81, 82). The catalytic reaction is activated when a CO_2_ molecule binds to the lysine in the active site and is stabilized with a Mg^2+^ ion (83, 84). The substrate ribulose-1,5-bisphosphate (RuBP, a five-carbon sugar) binds to the carbamate, forming a six-carbon intermediate that is then bound to a second CO_2_ and converted to two 3-phosphoglycerate (3-PGA, a three-carbon sugar) molecules, critical metabolites for the Calvin Cycle (85).

While rubisco was rapidly identified as the signature enzyme inside of the α-CB (7), its organization inside the α-CB was considerably more difficult to resolve. The first rubisco density resolved within a α-CB was a low resolution (~40 Å) subtomogram average (STA) within the α-cyanobacteria *Synechococcus* WH8109 cells (54). While the resolution was too low to draw structural conclusions, the authors observed concentric layers on the interior, suggesting some sort of enzyme organization (54). The cargo also clustered in partial α-CBs, suggesting either concurrent encapsulation and shell assembly processes or cargo attachments to the shell that persist if the α-CB is broken (54).

**Fig 5:**
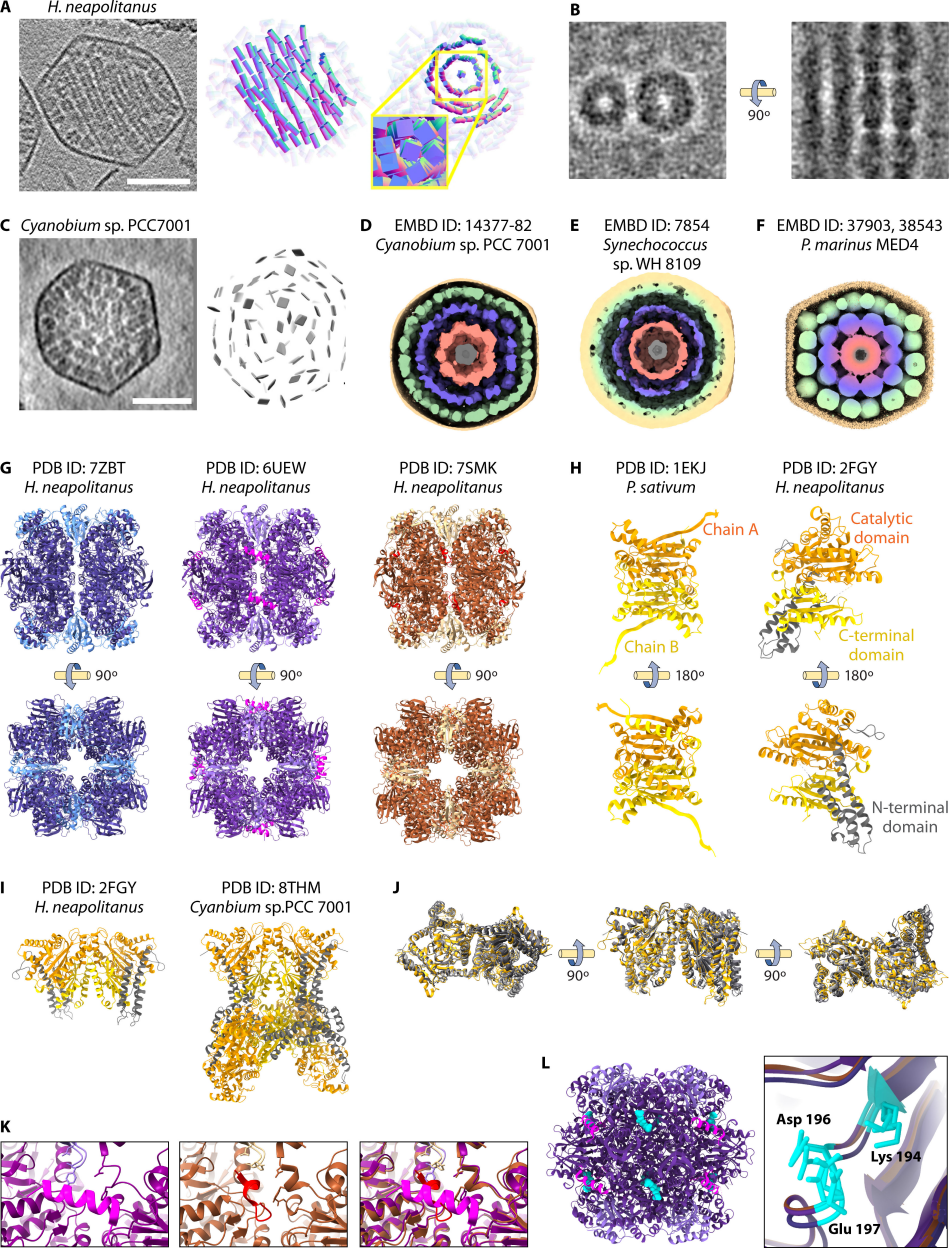
Enzyme organization within α-CBs. (**A**) *H. neapolitanus* structures reveal rubisco organized in fibrils. Representative micrograph and model of particle arrangement and 2D projection of rubisco particles in fibrils published previously in Metskas, et al., 2022 (55). (**B**) Representative orthoslice of a tomogram published previously in Metskas, et al., 2022 (55) reveals the spacing between fibrils. (**C**) *Cyanobium* sp. PCC 7001 structures reveal rubisco organized in concentric circles. Representative micrograph and model of particle arrangement published previously in Ni, et al., 2022 (42). This concentric ultrastructure has been observed with cryo-EM in the following species: (**D**) *Cyanobium* sp. PCC 7001 (EMD-14377-82) (53). (**E**) *Synechococcus* sp. WH8109 (EMD-7854) (54). (**F**) *Prochlorococcus* (EMD-37903, 38543) (24). (**G**) Recent structures of rubisco demonstrate the homogeneity of its structure. A cryo-ET structure of rubisco complex large and small subunits in an α-CB (blue, PDB ID: 7ZBT) (42); an x-ray structure of rubisco (purple) with a fusion CsoS2 peptide (magenta, PDB ID: 6UEW) (22); and a cryo-EM structure of purified rubisco (brown) incubated with the CsoSCA peptide (red, PDB ID: 7SMK) (8). (**H**) Comparison of the multichain symmetry of a typical β-class carbonic anhydrase dimer (PDB ID: 1EKG) (87), with the pseudosymmetry of the CsoSCA carbonic anhydrase (PDB ID: 2FGY) (38) showing the single catalytic domain, diverging C-terminal domain, and the N-terminal domain extension on a single chain. (**I**) Carbonic anhydrase structures from α-CBs. Top, dimer of pseudodimers from *H. neapolitanus* (PDB ID: 2FGY) (38); bottom, hexamer of pseudodimers from *Cyanobium* sp. PCC 7001 incubated with RuBP (PDB ID: 8THM) (88). (**J**) Dimers are aligned from 2FGY and 8THM to demonstrate secondary structure homogeneity; however, the dimer interface is inherently flexible even within the hexamer dimers. (**K**) Left, zoomed-in view of the CsoS2 peptide bound to rubisco in 6UEW. Middle, zoomed-in view of the CsoSCA peptide bound to rubisco in 7SMK. Right, both binding sites aligned to view in incompatible simultaneous binding. (**L**) Left, rubisco (PDB ID: 6UEW) (22) is aligned with a crystal structure incubated with RuBP (cyan, PDB ID: 1RXO) (81) to demonstrate the active sites compared to the sites for CsoS2 (blue) and CsoSCA (pink) binding from panel **G**. Right, key residues in the active site that bind to RuBP are aligned from structures shown in panel **C**.

Advances in cryo-ET techniques have provided key insights into rubisco organization and packing inside α-CBs, illustrated in several recent studies (55). STA of α-CBs isolated from *H. neapolitanus* revealed the wild-type rubisco organization (55) (Fig. 5A and B). Using 3D particle locations from their 4.5 Å rubisco structure to complete an ultrastructural model, the authors found that rubisco formed fibrils that organized into lattice structures with a sixfold pseudosymmetry inside a sub-population of α-CBs (Fig. 5A and B), a morphology that was verified using lower-resolution *in situ* tomography by the same study (55) and subsequently replicated (42). This order was not the predicted, concentric layers of the low-resolution *Synechococcus* studies (54); however, the rubisco first filled a layer immediately interior to the shell before occupying the center of the α-CB (55). While rubisco forms fibrils in crystal structures as well, the interface and lateral organization differed *in situ* (22, 86). The characterization of the fibrils in this study provided critical information about the cargo packing and rubisco-rubisco interface in a more native environment with all cso operon components at physiological concentrations (55).

Characterization of *Cyanobium* sp. PCC 7001 ultrastructure has also been published using purified α-CBs (24, 42, 53, 54) (Fig. 5C). The *Cyanobium* α-CB is smaller and more icosahedral than that of *H. neapolitanus*, likely related to its only containing a single isoform of CsoS2 (65). In contrast to the fibrils of *H. neapolitanus* α-CBs (42, 55), cryo-ET observed concentric rings of rubisco oligomers (42) (Fig. 5C). This concentric arrangement was validated using a single particle cryo-EM of intact *Cyanobium* α-CBs (53). Masking four internal layers, the individual layers were resolved to ~18 Å (Fig. 5D). The organization was homogeneous enough for symmetry expansion to improve the map. The averaged layers provide insights into the number of rubisco in each layer (192 outer layers, 72 middle layers, 32 inner layers, and 4 core layers for a count of ~300 per intact α-CB). Averaging of the shell structurally confirmed the icosahedral symmetry observed in other studies. Although the α-CBs of *Cyanobium* are homogenous in shell size and structure, high resolution was still not attained. This finding suggests that asymmetry in the structure or composition remains. Additionally, the workflow of single-particle cryo-EM necessarily includes classification steps, eliminating outliers, resulting in a final structure that is a subset of the *Cyanobium* shell architecture. Single particle cryo-EM has observed similar morphologies in *Synechococcus* sp. WH8109 (54) (Fig. 5E) and *Prochlorococcus* MED4 (24) (Fig. 5F).

The interactions of rubisco with other components of the α-CB provide possible mechanisms to explain these heterogeneities. Current structures of rubisco from α-CBs are homogenous (8, 22, 42) but reveal interfaces for several different structural proteins of the α-CB, including CsoS2 (22) and CsoSCA (8) (Fig. 5G).

### Carbonic anhydrase (CsoSCA)

CsoSCA, previously known as CsoS3 (38), is a subclass of β-carbonic anhydrases. This enzyme catalyzes the interconversion of bicarbonate and CO_2_ to equilibrium (38, 89). Interestingly, the *csoSCA* sequence diverges so strongly from other known carbonic anhydrase classes that its function was determined through knockouts and biochemical assays (38). Structurally, CsoSCA comprises an N-terminal domain responsible for α-CB encapsulation, a middle domain containing catalytic and zinc-binding sites, and a C-terminal domain with an unknown function (38, 88).

Due to its unusual structure, CsoSCA was initially identified as a novel lineage of carbonic anhydrases (90). However, the crystal structure of *H. neapolitanus* (*Hn*) CsoSCA (PDB ID: 2FGY) (38) showed structural homology to the β-class carbonic anhydrases with several exceptions. Most known β-carbonic anhydrases are symmetric as they are a homodimer formed by two catalytic domains on separate polypeptide chains with one active site each (91). In contrast, *Hn*CsoSCA contains pseudosymmetry between two diverging domains on a single polypeptide chain due to an apparent gene duplication event (38) (Fig. 5H). *Hn*CsoSCA conserves a functional catalytic domain with carbonic anhydrase activity at a zinc-binding site (38). However, despite being weakly homologous to the catalytic domain, the C-terminal pseudosymmetry partner lacks this zinc-binding site and thus lacks carbonic anhydrase activity (38). This study also identified a novel N-terminal domain in *Hn*CsoSCA that is not present in other β-carbonic anhydrases (38).

The structure of the RuBP-dependent *Cyanobium* sp. PCC 7001 (*Cy*) CsoSCA by x-ray crystallography (PDB ID: 8THM) provided additional insights into its structure, regulation, and oligomerization in α-cyanobacteria (88). This structure identified binding of the rubisco substrate RuBP in a *Cy*CsoSCA hexamer (trimer of dimers) (88) (Fig. 5I). The *Cy*CsoSCA RuBP-binding site is positioned near the CTD and dimer interface, allosterically activating the *Cy*CsoSCA (88). Comparison of the previously solved dimer (*Hn*CsoSCA) and hexamer (*Cy*CsoSCA) structures shows overall secondary structure conservation but heterogeneity of the position of the second chain in each dimer (Fig. 5J). Importantly, since the initial description of *Hn*CsoSCA as a dimer of pseudodimers in solution (38), more recent studies have found that like *Cy*CsoSCA, *Hn*CsoSCA is also a hexamer of pseudodimers in solution (8, 88); this discrepancy is attributed to a mutated N-terminal domain that prevented formation of the hexamer in the original structure (88).

CsoSCA regulation is poorly understood and may differ between organisms. *Hn*CsoSCA is constitutively active (38), while *Cy*CsoSCA is activated by the rubisco substrate RuBP (88) (Fig. 5F). Evaluations of CsoSCA activity in the presence of other small molecules and pH ranges have also been performed (88), but contextualization of these results is hampered by a dearth of knowledge for chemical conditions in the α-CB lumen. Several sulfur chemolithotrophs with α-CBs lack the classical β-class carbonic anhydrase and instead express an ι-class carbonic anhydrase, possibly an adaptation to alkaline growing conditions with low cofactor solubility (76).

While initial studies in *H. neapolitanus* predicted a direct CsoSCA-shell interaction based on anti-CsoSCA antibodies localizing near the shell in thin-section TEM (90, 92), a recent study found that the N-terminal domain bound to rubisco, not the shell, with this interaction required for α-CB encapsulation of CsoSCA (8). The binding results were ultimately confirmed with a high-resolution cryo-EM structure of rubisco in complex with CsoSCA residues 1–50, which showed CsoSCA residues P22-A30 binding a pocket between the two large subunits of rubisco (Fig. 5G). While the CsoSCA C-terminal domain may mediate interactions with rubisco or the α-CB shell, its function, if any, remains unknown.

### Rubisco as a hub for encapsulation in α-CBs

The structural interactions of CsoSCA and CsoS2 with the rubisco complex provide insights into the encapsulation process. Similarly to CsoSCA, x-ray crystallography recently revealed that the N-terminal domain of CsoS2 multivalently binds to the rubisco complex at 200 nM concentration, with the four repeats having varying binding affinities. The resulting structure identified eight binding sites for CsoS2 on the outer surface of rubisco (22) (Fig. 5G and K). It has previously been suggested that the presence or absence of multiple CsoS2 isoforms plays a critical role in determining the rubisco organization within the α-CB (42). Additionally, intrinsically disordered proteins are often capable of phase separation, and multivalent binding can allow a folded protein to join the condensate, suggesting a potential mechanism for cargo condensation through liquid-liquid phase separation concurrent with α-CB shell assembly (22). Rubisco condensation is seen in other CCMs including the cyanobacterial β-CB (25) and the eukaryotic pyrenoid (93).

Interestingly, CsoS2 binds rubisco in the same pocket as CsoSCA (8) (Fig. 5K). The CsoSCA-binding site is deeper within the same pocket as the CsoS2-binding site, and full CsoS2 occupancy on rubisco eliminated CsoSCA binding (8). This suggests that the cargo encapsulation process is dynamic, with different intermolecular interactions working in concert (8). This binding pocket is not near the active site where RuBP binds (Fig. 5L). When aligning the unbound, CsoS2-bound, and CsoSCA rubisco structures, the active site appears largely unchanged (Fig. 5L), suggesting binding to these accessory proteins does not affect the activity.

## PERIPHERAL α-CB PROTEINS

**Fig 6:**
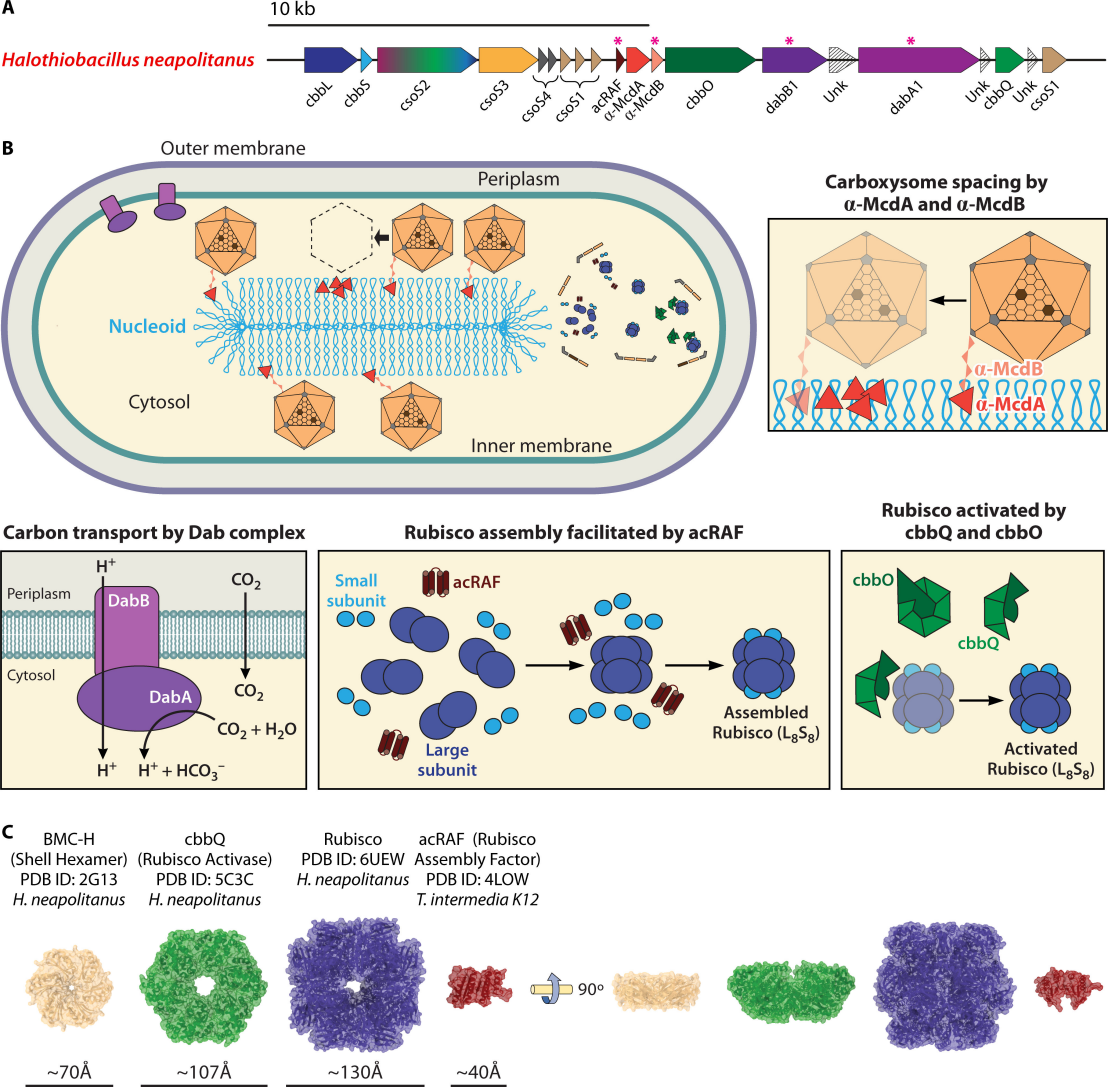
Peripheral α-CB proteins. (**A**) Adapted from Zhou et al. 2024 (24), updated annotated *H. neapolitanus* cso locus and surrounding areas highlighting recently identified genes implicated in α-CB function. (**B**) Modeled roles for peripheral α-CB proteins in *H. neapolitanus* include the Dab inorganic carbon transporter complex, the α-carboxysome rubisco assembly factor (acRaf) rubisco assembly factor, rubisco activase activity of a CbbQ/CbbO complex, and a Maintenance of Carboxysome Distribution proteins A and B (McdAB) CB positioning complex. (**C**) Comparison of the structures of the BMC-H hexamer (PDB ID: 2G13) (57), CbbQ rubisco activase hexamer (PDB ID: 5C3C) (94), rubisco hexadecamer (PDB ID: 6UEW) (22), and acRaf rubisco assembly factor of *H. neapolitanus* (PDB ID: 4LOW) (45).

The cso operon is part of a locus broadly associated with the CCM activity and implicated in α-CB function (Fig. 6A). The structures of these proteins provide additional insights into CCM processes in these organisms and suggest that the α-CB shell may be a dynamic enzyme-associated structure rather than a passive semipermeable barrier as formerly thought (61, 62). While heterologous expression of the cso operon without these proteins is sufficient to produce carbon-fixing α-CBs in new hosts (67), these are possible mechanisms for increasing the yield or activity of the broader CCM.

### Rubisco complex formation and reactivation (acRaf, CbbQ/CbbO)

The formation of a functional rubisco complex typically requires chaperones and assembly factors (95). These pathways have been well documented in cyanobacterial β-CBs. Rubisco large subunit folding is controlled by a GroEL-ES chaperone system (96). The assembly factors Rac1 (97) and RbcX (98) modulate the formation of the rubisco hexadecamer complex and the subsequent formation of ordered rubisco condensates continuing throughout the lifetime of the β-CB (98, 99).

In contrast, these processes are relatively unknown in α-CB-expressing bacteria (24). acRAF is conserved downstream of the cso operon (24, 45) and has been suggested to stabilize formation of the hexadecamer complex in a similar manner to RbcX (45) (Fig. 6B). acRAF deletion results in decreased growth at ambient CO_2_ concentrations (28). The crystal structure of acRAF showed a BMC domain-like fold and an active catalytic site with an unknown mechanism of action (100) (Fig. 6C). Mass spectrometry studies have not detected acRAF in purified α-CBs (24, 35), so acRAF function may occur in the cytoplasm prior to α-CB assembly.

Once the fully folded rubisco complex is formed, rubisco activases maintain the activity of the complex (95). Rubisco activases contain a conserved AAA+ domain that disrupts the rubisco-binding site and releases an inhibitory sugar through ATP hydrolysis (95). In α-CBs, this activity is thought to be performed by either CbbX (44) in α-cyanobacteria or the CbbQ/CbbO complex in proteobacteria (43, 101, 102) (Fig. 6B). Despite diverging in their sequence (94), both CbbX and CbbQ/CbbO contain characteristic AAA+ domains and form a hexameric structure with a 25 Å or 19 Å pore that interacts with the disordered C-terminus of the large rubisco subunit (44, 94) (Fig. 6C) in a specific manner (43). The CbbQ/CbbO complex has been detected in the shell of both purified (35, 94) and recombinant (103) α-CBs at the expected ~6:1 CbbQ:CbbO ratio. The presence of rubisco activase activity in intact α-CBs implies an ATP flow across the shell barrier, which has not yet been modeled (20). As the CbbO/CbbQ of *H. neapolitanus* does not appear to be critical for α-CB function inside the cell (13) or for the activity of heterologously expressed α-CBs (28), a recent study has proposed that this activity is required only under certain conditions, dependent on the kinetics of inhibition of the encapsulated rubisco or the availability of energy and CO_2_ in the system (43).

### CB positioning and cellular organization (McdAB)

α-CBs are positioned throughout the cell to prevent aggregation and allow for efficient distribution between daughter cells (46, 104). Recently, an ortholog of the McdAB complex has been implicated as a critical component in this process in proteobacteria such as *Halothiobacillus neapolitanus* (46). This complex is composed of a ParA-type ATPase (α-McdA) that reversibly binds to the nucleoid and a disordered linker (α-McdB) that interacts with both the α-CB and α-McdA (46) (Fig. 6B). Using this complex, proteobacterial α-CBs are theorized to distribute via a Brownian ratchet model, but the mechanics of this process remain unclear (46, 104). This complex is absent in α-cyanobacteria (105).

In contrast to the McdAB complex of proteobacterial α-CBs, the orthologous McdAB complex of β-cyanobacteria has been better characterized (105–109), providing possible insights into structural mechanisms of this complex in proteobacterial α-CBs. In β-cyanobacteria, β-McdA visibly oscillates between cell poles via nonspecific ATP-driven binding to the nucleoid (106, 109). β-McdB is a largely disordered protein (109) that undergoes liquid-liquid phase separation (105), oligomerizes (46), and contains a C-terminus with conserved tryptophan residue that associates with β-CBs (107) and a charged N-terminus that interacts with β-McdA (109). β-McdB is thought to stimulate ATP-bound β-McdA to dissociate and relocate the CB-bound complex along the nucleoid (104, 109). However, nucleoid compaction has recently been suggested to also contribute to β-CB positioning, suggesting that this process is an emergent phenotype from a network of cellular signals (108).

The α-CB McdAB ortholog diverges in several key features from the McdAB of the β-CB. α-McdAB complexes are widespread in proteobacteria such as *Halothiobacillus neapolitanus*, where an McdAB ortholog is expressed in close proximity to the cso operon (46) (Fig. 6A). Disruption of this complex results in aggregation of α-CBs and abnormal cellular morphologies (46). While the α-McdA ortholog retains features of β-McdA orthologs, the α-McdB is significantly more disordered and does not oligomerize, but retains the C-terminal tryptophan sequence, charged N-terminus, and liquid-liquid phase separation activity (46, 107). Fascinatingly, an additional McdB ortholog is found removed from the cso locus in proteobacteria that lacks both an α-McdA partner and the charged N-terminus (46); the functional implications of these differences for spatial dynamics of α-CBs, the structure of these proteins, or to the function and regulation of this complex remain a topic of ongoing study (46, 104).

### Inorganic carbon pumps (DabAB)

Effective CCM function requires a cytosolic pool of bicarbonates (12). In both α- and β-cyanobacteria, inorganic carbon is taken up from the environment using a diverse suite of transporters (12). The diversity of these systems is thought to enable carbon fixation in the more dynamic conditions experienced by cyanobacteria (12, 19). In contrast, proteobacteria such as *Halothiobacillus neapolitanus* are thought to express several paralogs of a single transporter, encoded downstream of the cso operon (19) and designated the DAB (DABs accumulate bicarbonate) operon. The transporter is a complex composed of DabA and DabB, thought to form a transmembrane complex with carbonic anhydrase activity that uses the proton motive force to convert diffused CO_2_ into bicarbonate (13).

### Protein with unknown function (Ham1)

The locus of the cso operon frequently contains satellite genes with unknown function (24). Ham1 is present in low abundance in purified α-CBs and contains a putative histone acetyltransferase domain (24, 76). Alkaline proteobacteria lack β-carbonic anhydrase activity but retain the N-terminal region in “CsoSX,” mutation of which impacts α-CB formation (76).

## DISCUSSION AND FUTURE DIRECTIONS

Our understanding of α-CBs has advanced significantly since they were initially identified as polyhedral inclusion bodies within *Thiobacillus neapolitanus* (7, 48). Recently published structures have identified key interfaces, shell assembly and organization, and ultrastructures of cargo within the compartment. These studies have opened the field to questions regarding the assembly, encapsulation, regulation, maintenance, and disassembly. At the same time, parallel studies in β-CBs have been revealing new insights that may generate new areas of study in α-CBs (29, 88). Finally, these advances have highlighted the importance of non-cso proteins in the function of the α-CB.

The structures of the shell proteins raise several important questions. The tight hexamer packing with pores electrostatically favorable for passage of small anions raised the possibility that the α-CB shell is a passive, selectively permeable barrier specific to passage of substrates and products (57, 63). This model was further supported by the presence of multiple paralogs of BMC domains with different pore properties (64, 72). Finally, biosensor-based studies of recombinant α-CB lumens showed a more acidic pH than the cytosol (14) and increased encapsulated enzymatic thermostability (41), suggesting a role for the α-CB shell in creating an optimized chemical environment for its contents. However, these measurements are indirect, and modeling of shell permeability suggests it would not be impenetrable to untargeted small molecules (59, 60, 63). Combined with recent insights into peripheral α-CB proteins, these findings suggest a more nuanced role of the shell in contributing to the activity of the α-CB.

The efforts to study α-CB ultrastructure through purified samples of the entire operon have been critical for identifying differences across α-CB-containing species (24, 53, 55, 73). However, the heterogeneity observed has led to new questions. Structures to date have predominantly been of samples that are devoid of cellular context, either through recombinant expression or computational isolation of small populations. As tomography technology and processing techniques improve, future efforts to resolve these compartments in their native cellular context are needed to answer questions about assembly and regulation.

In addition, there are poorly defined functions of domains in the cargo proteins. The CsoS2 middle domain appears to control the curvature; however, these observations are complicated by the diversity of CsoS2 repeats across species (23, 78). The short form of CsoS2 in *H. neapolitanus* has shown encapsulation into the shell can occur when all other cargo proteins are absent (23). This observation, along with the cryo-EM structures, has validated M-repeat interactions with the shell proteins (24, 73). Very early studies proposed that rubisco could interact with the M-repeats (74), but recent studies have not found evidence of this; however, binding studies have not been conducted under biological concentrations and chemical conditions, which may impact transitory or weak interactions.

The carbonic anhydrase is perhaps the least understood α-CB component. Whether the C-terminal domain of CsoSCA contributes to α-CB function is unknown. An additional mystery is the regulation of enzyme activity and organization inside the α-CB, whether it is shell-associated as previously thought (90, 92) has been recently questioned (8); however, the conditions of *in vitro* assays may not fully replicate biological conditions.

Finally, insights from studies in β-CBs can inform potential converging or diverging processes in the understudied life cycle of α-CBs. The larger size of β-CBs has facilitated studies into their assembly mechanism (110), including rubisco chaperone activity (99), response to changing cellular oxidative states resulting in exposure to reactive oxygen species (111), and deactivation/disassembly (29). These processes are poorly understood for α-CBs but likely occur through diverging mechanisms given the extensive evolutionary and morphological divergences between α-CBs and α-CBs (20). Future studies are required to elucidate these processes.

Insights from these studies in α-CBs will impact future efforts into the bioengineering of microcompartments for novel uses (20). Recombinant expression of α-CBs is currently being explored for synthetic bioreactors such as expressing chimeric shells (6, 58), heterologous expression in plants to improve yield (112, 113), or the expression of shells with custom targeted cargo (41, 114). These emerging applications draw from millions of years of evolutionary history to address a diverse array of current issues.
